# Specific Surface Modifications of Silica Nanoparticles Diminish Inflammasome Activation and *In Vivo* Expression of Selected Inflammatory Genes

**DOI:** 10.3390/nano7110355

**Published:** 2017-10-30

**Authors:** Viviana Marzaioli, Christina J. Groß, Ingrid Weichenmeier, Carsten B. Schmidt-Weber, Jan Gutermuth, Olaf Groß, Francesca Alessandrini

**Affiliations:** 1 Center of Allergy and Environment (ZAUM), Technical University and Helmholtz Center Munich, Member of the German Center for Lung Research (DZL), Ingolstädter Landstr. 1, 85764 Neuherberg, Germany; viviana.marzaioli@gmail.com (V.M.); ingrid.weichenmeier@gmx.de (I.W.); csweber@tum.de (C.B.S.-W.); 2 Institute for Clinical Chemistry and Pathobiochemistry, Klinikum rechts der Isar, Technical University of Munich, 81675 Munich, Germany; christina.thomas@tum.de (C.J.G.); olaf.gross@tum.de (O.G.); 3 Vrije Universiteit Brussel (VUB), Universitair Ziekenhuis Brussel (UZ Brussel), Department of Dermatology, Laarbeeklaan 101, 1090 Brussels, Belgium; Jan.Gutermuth@uzbrussel.be; 4 Institute of Neuropathology, Medical Center—University of Freiburg, 79106 Freiburg, Germany

**Keywords:** silica nanoparticles, inflammation, inflammasome, *in vitro* vs. *in vivo*

## Abstract

Silica (SiO_2_) nanoparticles (NPs) usage includes, but is not limited to, industrial and biomedical applications. Toxic effects of SiO_2_ NPs have been explored either *in vitro* or *in vivo*, assessing different surface modifications to reduce their harmful effects. Here, murine bone marrow-derived dendritic (BMDC) and a mouse model of mild allergic inflammation were used to study inflammasome activation and lung inflammation. Our results showed that SiO_2_ plain NPs induced NACHT, LRR and PYD domains-containing protein 3 (NLRP3) inflammasome activation, increasing interleukin (IL)-1β release *in vitro*, and, to a lesser extent, *in vivo*. In addition, SiO_2_ plain NPs triggered a pulmonary inflammatory milieu in both non-sensitized (NS) and sensitized (S) mice, by inducing the expression of key inflammatory cytokines and chemokines. Electron microscopy showed that SiO_2_ NPs were mostly localized in alveolar macrophages, within vesicles and/or in phagolysosomes. Both the *in vitro* and the *in vivo* effects of SiO NPs were attenuated by coating NPs with phosphonate or amino groups, whereas PEGylation, although it mitigated inflammasome activation *in vitro*, was not a successful coating strategy *in vivo*. These findings highlight that multiple assays are required to determine the effect of surface modifications in limiting NPs inflammatory potential. Taken together, these data are obtained by comparing *in vitro* and *in vivo* effects of SiO_2_ NPs suggest the use of amino and phosphonate coating of silica NPs for commercial purposes and targeted applications, as they significantly reduce their proinflammatory potential.

## 1. Introduction

Commercial use of nanoparticles (NPs) has been increasing over the last 10 years, prompting a new demand for safety investigation on the use of nanoparticles. The small size of 100 nm or less confers NPs unique physical and chemical characteristics, and a high degree of versatility in industrial use [1]. However, the same property increases their penetration into the body, in particular through respiration [2,3], leading to lung accumulation of NPs [4,5]. For example, very recently, the European Chemicals Agency has announced that TiO_2_ NPs should be classified as suspected of causing cancer through the inhalation route [6]. Silicon dioxide (silica [SiO_2_]) nanoparticles are widely used in industrial applications as an additive for rubber and plastics, and strengthening filler for concrete. In addition, they are used in biomedical applications for drug delivery and theranostics [7,8,9]. In the last few years, an increasing body of literature investigated the possible harmful effects following SiO_2_ NP exposure. *In vitro*, SiO_2_ NPs has been shown to induce oxidative stress, platelet aggregation, and cell death [10,11,12,13,14,15]. In addition, SiO_2_ and TiO_2_ NPs have been shown to induce inflammasome activation in murine dendritic cells [16]. Activation of the inflammasome has been associated with lung fibrosis and toxicity [17,18], and it has also been shown to be the main event in the development of lung fibrosis following chronic inhalation of crystalline silica dusts [19].

*In vivo*, it has been shown that submicron amorphous silica particles have greater inflammatory properties and cytotoxicity than bigger particles [20]. In addition, we and others have shown that acute and chronic exposure to SiO_2_ NPs aggravate airway inflammation [21,22,23,24,25].

The use of surface modifications to coat nanoparticles is an emerging strategy to try to “mask” the toxic effects of nanoparticles, and it has been shown to reduce aggregation and nonspecific binding of NPs [26]. *In vitro* studies have shown that the surface modification of SiO_2_ NPs reduces their potential for inflammasome activation and cytotoxicity [27,28]. In addition, we have previously shown that surface modification diminished the proinflammatory and immunomodulatory effect of SiO_2_ NPs in a murine ovalbumin (OVA)-induced model of allergic airway inflammation [21]. 

This study aims to investigate and compare both *in vivo* and *in vitro* effects of SiO_2_ NP surface modifications. Our data showed inflammasome activation in murine bone marrow-derived dendritic cells (BMDC) by SiO_2_ plain NPs, which was strongly diminished with the use of phosphonate (-P) and amino (-NH_2_) group surface modifications of SiO_2_ NPs. *In vivo*, SiO_2_ plain NPs did not activate the inflammasome in non-sensitized mice (NS), but, IL-1β and caspase 1 mRNA expression were increased in sensitized (S) mice that were instilled with PEGylated (-PEG)SiO_2_-NPs. Again, this effect was diminished in sensitized mice instilled SiO_2_-P and SiO_2_-NH_2_. In addition, amino and phosphonate surface modification, but not PEGylation, markedly decreased the expression of key inflammatory genes in the lung of both NS and S mice that were intratracheally instilled with SiO_2_ plain NPs. These diverging results obtained from *in vitro* and *in vivo* data underline the importance of comparing test results from different assays to evaluate the biological effects and potential health hazards of silica NPs with different surface modifications.

## 2. Results

### 2.1. Surface Modifications of Silica Nanoparticles Diminish IL-1β Release

**Figure 1:**
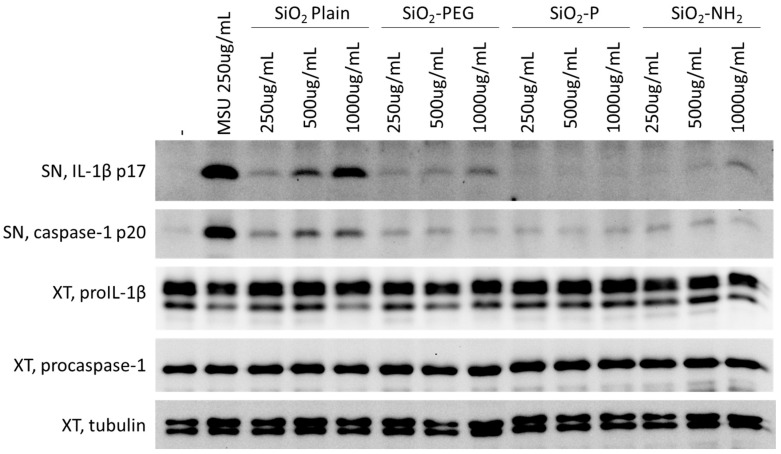
Surface modifications of silica nanoparticles diminish IL-1β release. Western blotting for IL-1β active form (p17) or pro-form (pro IL-1β) in supernatants (SN) or cellular extracts (XT) and cleaved caspase-1 (p20) in supernatant or pro-form in XT (pro-caspase-1) in LPS-primed murine BMDC treated with increasing concentration (250–1000 µg/mL) of plain SiO_2_, SiO_2_-PEG, SiO_2_-P or SiO_2_-NH_2_. Monosodium urate (MSU) crystals were used as positive control and tubulin as housekeeping gene for loading control in XT.

To evaluate the possible effect of surface modification of silica nanoparticles on the activation of the inflammasome, IL-1β secretion was evaluated in lipopolysaccharide (LPS)-primed murine bone marrow-derived dendritic cells (BMDC) (Figure 1). Cells were stimulated with increasing concentration (250–1000 µg/mL) of uncoated SiO_2_ (SiO_2_ plain), PEGylated SiO_2_ (SiO_2_-PEG), phosphonate-coated SiO_2_ (SiO_2_-P), or amino-coated SiO_2_ (SiO_2_-NH_2_). NPs and the presence of the cleaved form of IL-1β (IL1-β p17) in the supernatant was measured by immunoblot, as described before [29]. Consistent with previous reports using silica from other sources [15], we found that SiO_2_ plain induced dose-dependent secretion of IL-1β into the supernatant. Interestingly, this induction was much lower in cells treated with SiO_2_-PEG, and was completely absent in cell treated with SiO_2_-P or SiO_2_-NH_2_, suggesting that these modifications reduce the capacity of the particles to engage the cellular processes involved in IL-1β production, potentially by interfering with inflammasome activation. Similar results were observed for the cleaved form of caspase-1, although its induction by SiO_2_ plain NPs was less than the one observed for IL-1β. Monosodium urate (MSU) crystal were used as a positive control [30].

### 2.2. Surface Modifications of Silica Nanoparticles Diminish Inflammasome Activation

**Figure 2:**
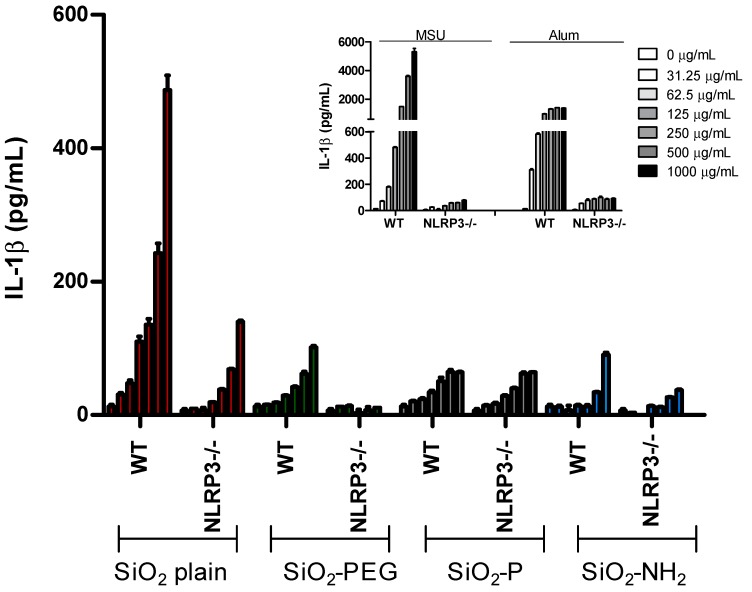
Surface modifications of silica nanoparticles diminish inflammasome activation. ELISA for IL-1β from WT or NLRP3^−/−^ murine bone marrow-derived dendritic cells (BMDC) treated with increasing concentration (0–1000 µg/mL) of SiO_2_ plain (red), SiO_2_-PEG (green), SiO_2_-P (gray), SiO_2_-NH_2_ (blue). As control Alum or Monosodium Urate Crystals (MSU) were used (inset).

In order to establish whether the IL-1β modulation observed by our silica NPs was dependent on the activation of the inflammasome, we measured IL-1β release from wild type (WT) or NLRP3^−/−^ murine BMDC treated with increasing concentrations (0–1000 µg/mL) of SiO_2_ NPs by enzyme-linked immunosorbent assay (ELISA) (Figure 2). In agreement with the increased protein expression observed in supernatant, we found a dose-dependent increase of IL-1β released from WT cells stimulated with SiO_2_ plain NPs (in red). This effect was decreased in cells lacking NLRP3, confirming that SiO_2_ plain NPs activate this inflammasome sensor protein [15].

In agreement with the IL-1β signal observed by immunoblotting, SiO_2_-PEG NP stimulation led to a lower induction of IL-1β (in green), which was further decreased in cells lacking NLRP3. In agreement with the immunoblot data, SiO_2_-P (in gray) or SiO_2_-NH_2_ (in blue) NP stimulation induced only a minimal IL-1β signal. Together, these data suggest that coating of silica NPs with -P or -NH_2_, and, to a lesser extent, PEG, reduces the ability of these particles to activate the NLRP3 inflammasome.

### 2.3. Surface Modifications of Silica Nanoparticles Diminish Selective Inflammatory Genes Expression In Vivo

**Figure 3:**
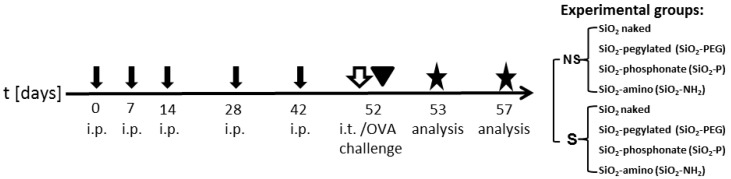
Experimental protocol. BALB/c mice were intraperitoneally sensitized (S) with OVA/aluminum hydroxide or non-sensitized (NS) with phosphate-buffered saline/aluminum hydroxide (black arrows). On day 52, S and NS mice were intratracheally instilled with SiO_2_ nanoparticles or with CTL supernatant (white arrow) and subsequently challenged with OVA aerosol (arrowhead). Gene expression in pulmonary tissue was analyzed on day 57. Experimental groups are listed.

Having established the ability of surface modifications to dampen the activation of the inflammasome by SiO_2_ NPs, we sought to evaluate the effect of silica NPs and their surface modification *in vivo*. Mice were intraperitoneally sensitized (S) with OVA/aluminum hydroxide or left non-sensitized (NS) by injection of phosphate-buffered saline/aluminum hydroxide as a control for a total of six weeks. 10 days after the last injection, mice were intratracheally instilled with SiO_2_ nanoparticles or with supernatant control (CTL) and subsequently challenged with OVA aerosol. Gene expression in the lung was evaluated after five days from the OVA-challenge/instillation (Figure 3). 

**Figure 4:**
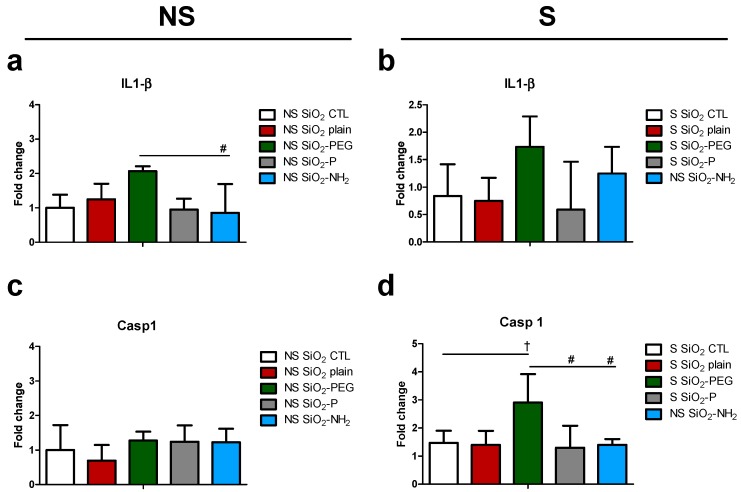
Effect of SiO_2_ NPs on inflammasome activation *in vivo*. IL-1β and Casp1 gene regulation in lungs of NS (**a**,**c**) and S mice (**b**,**d**) 5 days after intratracheal instillation of 50 μg SiO_2_ plain (red), SiO_2_-PEG (green), SiO_2_-P (gray) or SiO_2_-NH_2_ blue) or supernatant control (CTL) and ovalbumin challenge. Mean ± standard deviation were evaluated in the lung by real-time PCR (*n* = 4/group). One-way Anova † *p* < 0.05; vs. CTL. # *p* < 0.05; vs. SiO_2_-PEG (*n* = 4).

The analysis of gene expression in the lungs of NS mice showed that SiO_2_ plain NPs did not induce an increase of IL-1β expression (Figure 4a); in addition, also caspase 1 (Casp1) a key component of the inflammasome and the protease cleaving IL-1β to produce its mature, bioactive form, and controlling its secretion was not modulated (Figure 4c). However, SiO_2_-PEG instillation lead to an increase in IL-1β expression that was not, or to a lesser extent, observed with SiO_2_-NH_2_ and SiO_2_-P (Figure 4a). In S mice, however, IL-1β was increased, although not significantly in SiO_2_-PEG (Figure 4b). Interestingly, although Casp1 expression was not modulated in the lung of NS mice (Figure 4c), S mice showed a significant increase of Casp1 when instilled with SiO_2_-PEG. This effect was not observed in mice instilled with SiO_2_-NH_2_ and SiO_2_-P NPs (Figure 4d). To note is that the sensitization itself did not significantly modulate the expression of IL-1β and Casp1 (Figure S2a). 

**Figure 5:**
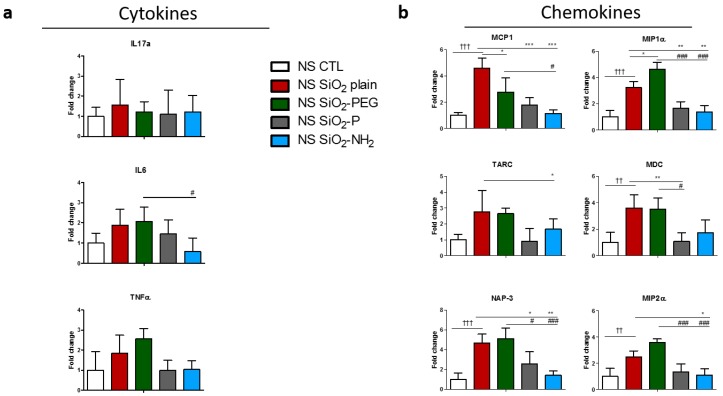
Surface modifications of silica nanoparticles diminish expression of certain inflammatory genes in non-sensitized mice (NS) mice *in vivo*. Representative gene regulations of (**a**) cytokines or (**b**) chemokines in mouse lungs 5 days after intratracheal instillation of 50 μg SiO_2_ plain (red), SiO_2_-PEG (green), SiO_2_-P (gray) or SiO_2_-NH_2_ blue) or supernatant control (CTL) and ovalbumin challenge. Mean ± standard deviation were evaluated in lung by real-time PCR (*n* = 4/group). One-way Anova † *p* < 0.05; †† *p* ≤ 0.01; ††† *p* ≤ 0.01 vs. CTL *p* ≤ 0.001; * *p* < 0.05; ** *p* ≤ 0.01; *** *p* ≤ 0.001 vs. SiO_2_ plain. # *p* < 0.05; ## *p* ≤ 0.01; ### *p* ≤ 0.001 vs. SiO_2_-PEG (*n* = 4). Part of the data, i.e., those relative to the cytokine TNF-α and to the chemokines MCP-1, MIP-1α, TARC and MDC have already been shown in a different form in [21].

We next sought to investigate the effect of SiO_2_ NPs on gene transcription of cytokines and chemokines involved in inflammation (Figure 5) in non-sensitized mice (NS) as a surrogate for healthy humans. IL-17 was not affected by SiO_2_ plain NPs. However IL-6 and tumor necrosis factor (TNF)-α were increased, although not significantly, in mice instilled with SiO_2_ plain and SiO_2_-PEG NPs. IL-6 expression, moreover, was significantly decreased in mice treated with SiO_2_-NH_2_, when compared to SiO_2_-PEG mice (Figure 5a). We then evaluated the transcription of chemokine genes in the lung of mice instilled with NPs (Figure 5b). Interestingly, monocyte chemotactic protein 1 (MCP1), macrophage inflammatory protein 1-alpha (MIP-1α), macrophage-derived chemokine (MDC), neutrophil-activating protein 3 (NAP-3), and macrophage inflammatory protein 2-alpha (MIP2-α) expressions were all significantly and strongly increased in mice treated with SiO_2_ plain and SiO_2_-PEG NPs, and their expression was strongly mitigated when mice were instilled with SiO_2_-P or SiO_2_-NH_2_ NPs. Thymus and activation regulated chemokine (TARC) expression was upregulated in mice instilled with SiO_2_ plain and SiO_2_-PEG NPs, although not at a significant level, and SiO_2_-NH_2_ NPs treatment resulted in a significant reduction in expression when compared to SiO_2_-PEG. All together, these data suggest that the silica surface modification SiO_2_-P and SiO_2_-NH_2_ prevented the *in vivo* upregulation of certain inflammatory genes observed with SiO_2_ plain and SiO_2_-PEG in non-sensitized mice.

We then analyzed cytokine/chemokines gene transcription in sensitized (S) mice (as a surrogate for asthmatic humans) that were instilled with silica NPs. The sensitization protocol induced a strong inflammatory milieu into the lungs of mice instilled with NPs supernatant, by significantly inducing the expression of MCP1, TARC, MDC, NAP-3, and MIP2α. However, no strong increase was observed for IL-17, IL-6, and TNFα (Figure S2a,b).

**Figure 6:**
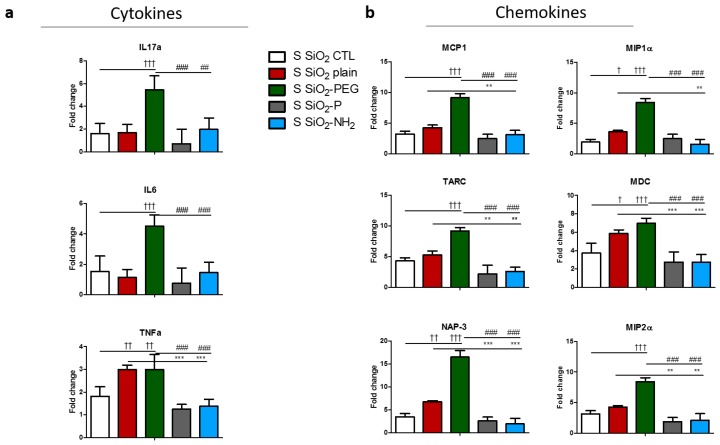
Specific surface modifications of silica nanoparticles diminish selective inflammatory genes expression in S mice *in vivo*. Representative gene regulations of (**a**) cytokines or (**b**) chemokines in mice lungs 5 days after intratracheal instillation of 50 μg SiO_2_ plain (red), SiO_2_-PEG (green), SiO_2_-P (gray) or SiO_2_-NH_2_ blue) or supernatant control (CTL), and ovalbumin challenge. Mean ± standard deviation were evaluated in lung by real-time PCR (*n* = 4/group). One-way Anova † *p* < 0.05; †† *p* ≤ 0.01; ††† *p* ≤ 0.01 vs. CTL *p* ≤ 0.001; * *p* < 0.05; ** *p* ≤ 0.01; *** *p* ≤ 0.001 vs. SiO_2_ plain. # *p* < 0.05; ## *p* ≤ 0.01; ### *p* ≤ 0.001 vs. SiO_2_-PEG (*n* = 4). Part of the data, i.e., those relative to the cytokine TNF-α and to the chemokines MCP-1, MIP-1α, TARC, and MDC have already been shown in a different form in [21].

IL-17 and IL-6 were significantly increased in mice instilled with SiO_2_-PEG NPs, but not in the one instilled with SiO_2_ plain (Figure 6a). SiO_2_-NH_2_ and SiO_2_-P NPs diminished both IL-17 and IL-6 expression in respect to SiO_2_-PEG NPs. Interestingly, TNFα expression was significantly increased in the lung of S mice instilled with both SiO_2_ plain and SiO_2_-PEG NPs, and was reduced in lungs instilled with SiO_2_-NH_2_ and SiO_2_-P (Figure 6a). Chemokine expression was also regulated in NPs-exposed S mice (Figure 6b). In particular, SiO_2_-PEG NPs induced the expression of all the chemokines tested, whereas SiO_2_ plain NPs significantly induced the expression of MIP1α, MDC, and NAP-3. For all of the chemokines tested, the instillation of SiO_2_-NH_2_ or SiO_2_-P NPs resulted in a diminished expression in respect to the inflammatory NPs (SiO_2_ plain or SiO_2_-PEG).

### 2.4. Intracellular Localization of Silica NPs Following Intratracheal Instillation

**Figure 7:**
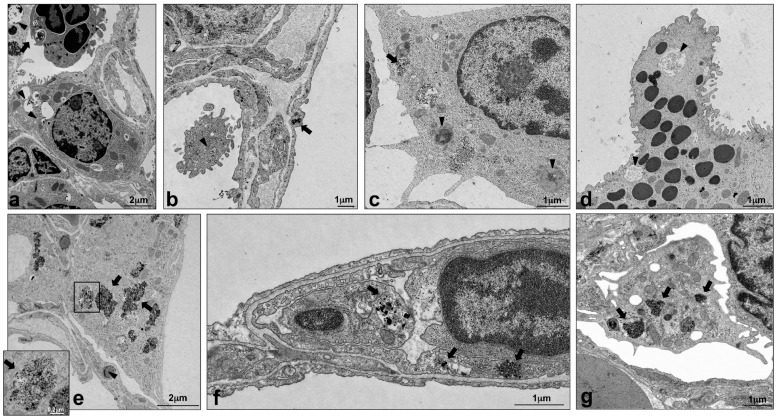
Localization of SiO_2_ NPs in the lungs by transmission electron microscopy. Representative pulmonary electron micrographs of NS (**a**,**b**,**f**,**g**) or S mice (**c**,**d**,**e**) exposed to SiO_2_ plain (**a**,**b**), SiO_2_-PEG (**c**,**d**), SiO_2_-P (**e**), and SiO_2_-NH_2_ (**f**,**g**). The analysis was performed 5 days after SiO_2_ NPs instillation and OVA challenge. Ultrastructural localization of SiO_2_ NPs (arrows) is evident in granulocytes (**a**), epithelial type I and type II cells (**a**,**b**), alveolar macrophages (**b**,**c**,**e**,**g**), endothelial cell (**f**). The particles were surrounded by a single membrane (arrow, inset in **e**). Phagolysosomes and/or autophagosomes (arrowheads) are evident in alveolar macrophages (**b**,**c**) and Clara cells (**d**).

In order to evaluate the localization of the instilled NPs in the lungs of S and NS mice, transmission electron microscopy (TEM) was used. Five days after intratracheal instillation, all four silica NPs were mainly localized within alveolar macrophages (Figure 7 partially in 7b, arrowhead; 7c, arrow and arrowheads; 7e, arrows; 7g, arrows); similar material with a different grade of agglomeration was found in smaller amounts also in alveolar epithelial cells (Figure 7a,b and Figure S1, arrows), endothelial cells (Figure 7f, arrows), and granulocytes (Figure 7a, arrow), confirming, as shown before [31], that SiO_2_ NPs can enter to a lower extent many different lung cell types other than macrophages. No difference in NPs localization was detected between S and NS mice. 

**Figure 8:**
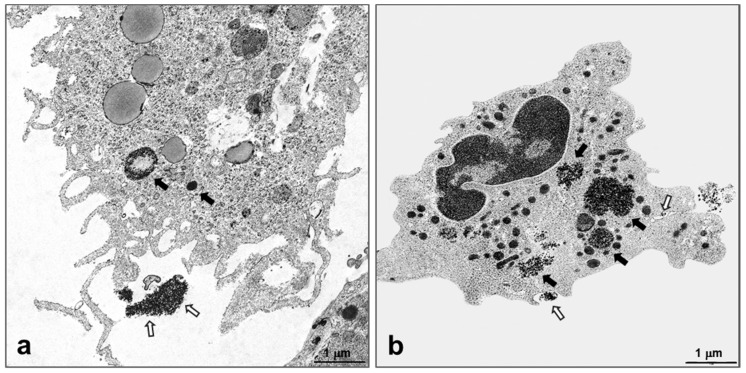
Localization of SiO_2_ NPs in the lungs by transmission electron microscopy. Representative electron micrograph of necrotic lung macrophage (**a**) and activated polymorphonuclear granulocyte (**b**) from NS lung exposed to SiO_2_ plain analyzed 5 days after SiO_2_ NPs instillation and OVA challenge. SiO_2_ NPs were present in vesicles within the cytoplasm (arrows) and outside of the cell (open arrows).

When NPs were found inside the cells, they were located in vesicles that were surrounded by a single membrane (Figure 7e, inset). The structure of the cells containing NPs appeared normal, although their cytoplasm was rich in particle-filled vesicles and phagolysosomes. Only in mice exposed to SiO_2_ plain, necrosis, or pyroptosis of macrophages occurred and polymorphonuclear granulocytes loaded with NPs were found in an activation state (Figure 8a,b, respectively). In addition, exclusively lungs exposed to SiO_2_ plain and SiO_2_ PEG presented hyperplastic Clara cells, a sign of injury to the main secretory cell of proximal and distal airways (Figure 7d, arrowheads showing autophagosomes). Lungs instilled with supernatant control displayed unchanged morphological appearance and, as expected, no particles could be detected in the cell cytoplasm (data not shown).

## 3. Discussion

This study demonstrated strong inflammasome activation in BMDC by SiO_2_ plain NPs *in vitro*, which was strongly diminished with the use of phosphonate (-P) and amino (-NH_2_) group surface modifications of SiO_2_ NPs. In contrast, *in vivo* SiO_2_ plain NPs did not activate the inflammasome in non-sensitized mice (NS), but, IL-1β and caspase 1 mRNA expression were increased by SiO_2_-PEG- instillation in sensitized and challenged (S) mice. Again, this activation was diminished by phosphate and amino surface modifications. Moreover, amino and phosphonate surface modification, but not PEGylation, strongly decreased the expression of key inflammatory genes in the lung of both NS and S mice mice that were intratracheally instilled with SiO_2_ plain NPs prior to allergen-challenge.

The broad use of NPs in industrial and biological contexts has led to an increasing body of literature aiming to evaluate potential harming effect of NPs in humans. A major limitation of most studies is their restriction either *in vitro* or *in vivo* systems to evaluate the effect of NPs, without providing a comparison of the two effects. The study presented here aimed to evaluate the pro-inflammatory effect of silica NPs and of different surface modifications in parallel *in vitro* and *in vivo* systems.

Inflammasomes play a critical role in early innate immune responses in the lung, but excessive inflammasome activation has also been associated with several pulmonary chronic inflammatory conditions [32]. Our data confirmed that SiO_2_ plain NPs activated the NLRP3 inflammasome *in vitro*. This is in agreement with previous studies [15], showing an increase of IL-1β by silica NPs in murine macrophage [33], dendritic cells [16], lung epithelial cells [34]**,** and in rat lungs [35]. 

In the attempt to investigate whether surface modifications could mitigate the effects of SiO_2_ plain NPs on the activation of the inflammasome, we used silica NPs with either PEG, phosphonate, or amino surface coating and demonstrated that PEG, phosphonate, and amino coating mitigated SiO_2_ plain inflammasome activation, by reducing IL-1β release by murine BMDC *in vitro*. This finding in amino- and phosphonate coated NPs is in line with previous *in vivo* studies that showed a reduction of lung inflammation in OVA-sensitized mice exposed to SiO_2_ amino or phosphonate [21]. 

We then extended our study to an *in vivo* setting in a mild allergic inflammation model [21,36]. We did not observe a significant upregulation of IL-1β mRNA after SiO_2_ plain NPs instillation in NS and S mice, however an increased tendency was observed after SiO_2_-PEG instillation. Caspase-1 was increased in S mice after SiO_2_-PEG instillation, but not SiO_2_ plain NPs, and this effect was mitigated by the inert surface modifications -P and -NH_2_, suggesting that, although PEGylation was a surface modification with inert effects *in vitro*, its effects were not confirmed *in vivo*. These results should also take into consideration the bigger aggregation, which might the affect particle uptake of NPs in medium (*in vitro*), in respect to water (*in vivo*), as previously characterized by Wohlleben and colleagues [37]. In addition, the *in vitro* studies are defined to one cell type, while *in vivo* SiO_2_-PEG can interact, and exert toxic effect, with different cell types within the lung. 

In NS mice we observed an increased tendency in TNFα and IL-6 expression after SiO_2_ plain and SiO_2_-PEG NPs instillation. IL-6 expression was reduced in mice instilled with SiO_2_-NH_2_, and to lesser extent also by SiO_2_-P NPs, when compared to mice instilled with SiO_2_-PEG NPs. This suggests that, although not statistically significant, PEGylated NPs exerted a pro-inflammatory effect in murine lungs of NS mice. However, in sensitized and allergen-challenged mice, SiO_2_ plain NPs increased the expression of TNFα, a key cytokine in inflammation, thus contributing to a more inflammatory milieu. SiO_2_-PEG NPs additionally induced IL-17 and IL-6 expression. All of these effects were diminished by -P and -NH_2_ surface modifications, supporting their inert role in inflammation, in agreement with previous studies [21,38,39]. 

Analysis of chemokine expression in NS mice showed a strong regulation of all chemokines tested, in particular by SiO_2_ plain NPs. This suggests that SiO_2_ plain induces a strong pulmonary pro-inflammatory micromilieu even in the absence of allergic sensitization, which recruits immune cells into the lung, in particular neutrophils (by NAP-3 and MIP2α [40]) and lymphocytes (by MDC and TARC [41,42]). SiO_2_ plain NPs thus elicited a mild degree of inflammation in non-sensitized mice and exacerbated the inflammatory response in the lungs of sensitized mice. The pulmonary recruitment of neutrophils and lymphocytes following silica NPs instillation was demonstrated in our previous study [21]. Interestingly, PEGylation of NPs did not mitigate the inflammation of the lung in both NS and S mice, as shown by the strong regulation of chemokines that was observed after SiO_2_-PEG instillation. This effect was observed in both our previous study, which analyzed broncho-alveolar cell infiltration, and in this current study, which analyzed lung inflammation on the molecular level. Here, we provide a mechanistic insight on the level of chemokine expression for the previously observed reduction of leucocyte infiltration [21] into the lung, where amino and phosphonate surface modifications induced a significant and strong reduction of pro-inflammatory and chemoattractant chemokines. 

Ultrastructural evaluation of lungs from both S and NS mice showed that silica NPs were localized in different cell types, but mainly in alveolar macrophages. NPs were contained in vesicles that were surrounded by a single membrane or in phagolysosomes, in agreement with a previous study with ultrafine carbon particles [43]. Autophagosomes have been previously shown to mediate lung inflammation via NLRP3 inflammasome signaling in macrophages [44,45]. Autophagosomes were detected in the cytoplasm of Clara cells (Figure 7d) and phagolysosomes in cells containing NPs (mainly alveolar macrophages, Figure 7a–c). Unfortunately, without a morphometrical analysis of autophagosomes in lung tissue, we could not observe if their frequency was enhanced in lungs exposed to SiO_2_ plain when compared to modified SiO_2_ NPS, and therefore cannot provide a correlation with the *in vitro* data. Necrotic lung macrophages were observed in lungs exposed to SiO_2_ plain, but not in lungs exposed to modified SiO_2_ NPs; it is likely that these macrophages had undergone pyroptosis, a process closely linked to inflammasome activation [46]. In addition, in the lungs exposed to SiO_2_ plain, activated neutrophils with cytoplasmatic protrusions and phagosomes containing particulate material (Figure 7a and Figure 8b) were observed, indicating that NPs activate and interact with neutrophils, as reviewed previously [47]. However, to certainly distinguish pyroptosis from other cell death processes, further analysis of cellular markers by flow cytometry are necessary. Underlining the proinflammatory properties of SiO_2_ plain and SiO_2_-PEG, hyperplastic Clara cells, which have been previously identified as a response of this protective cell type to stress [48] were only present in pulmonary airways exposed to SiO_2_ plain and SiO_2_-PEG and not in the airways of mice exposed to SiO_2_-NPs modified with -P and -NH_2_. A recent study might explain why PEGylation is not a successful strategy for mitigating NPs harmful effect [49]; it has been shown that the PEG high available surface area led to greater membrane interactions, which alone can induce macrophage activation [49]. It might also be possible that the PEG chain utilized in this study is too short for successfully coating the silica NPs. Previous studies have, in fact, shown that the PEG chain length is essential for determining the biodistribution of NPs [50]. We can speculate that -P and -NH_2_ coating interact differently with biological system, in respect to the -PEG or the plain NPs. Previous studies have, in fact, shown that surface modifications create a “corona” around the core of the NPs, which can strongly affect the interaction of the NPs with living systems [51]. Plain SiO_2_ and SiO_2_-PEG have a very similar corona and share up 70–80% of the corona proteins. In contrast, the oppositely charged SiO_2_ -P and -NH_2_ have different coronas, sharing only six proteins, as described by Wohlleben and colleagues [37]. Further studies are needed to clarify this hypothesis. 

Taken together, based on our *in vitro* and *in vivo* tests systems, amino- and phosphonate-coating represent inert surface modifications, which might have superior safety profile for exposed workers compared to SiO_2_ plain and SiO_2_-PEG NPs.

## 4. Materials and Methods

### 4.1. Nanoparticles Preparation

Uncoated SiO_2_ (SiO_2_ plain), PEGylated SiO_2_ (SiO_2_-PEG), phosphonate-coated SiO_2_ (SiO_2_-P), and amino-coated SiO_2_ (SiO_2_-NH_2_) NPs were obtained from BASF SE, Ludwigshafen, Germany. All of the NPs were amorphous and spherical in shape. The “SiO_2_ plain” material was surface modified to obtain the other three nanoforms of silica using covalently-bound PEG 500 (M_W_ = 500 g/mol), (trihydroxysilyl) propylmethylphosphonate (THPMP) and 3-aminopropyltriethoxysilane (APTES) for SiO_2_-PEG, SiO_2_-P and SiO_2_-NH_2_, respectively. Particle/agglomerate size was between 5 and 50 nm. Particle dispersion in water and in cell culture medium was measured by nanoparticle tracking analysis using a NanoSight LM20 system (Malvern, Herrenberg, Germany) [37]. The results of the D50 (nm) are the following: SiO_2_ plain 47 ± 16.9 and 188 ± 102; SiO_2_-PEG 54 ± 20.14 and 107 ± 64.7; SiO_2_-P 72 ± 43 and 137 ± 53.5; SiO_2_-NH_2_ 53 ± 22.5 and 145 ± 107.4 for water and medium, respectively. NPs were freshly diluted in water (aqua ad iniectabilia; B Braun Melsungen AG, Melsungen, Germany) and buffered with phosphate-buffered saline (PBS) to physiological pH, to the final concentration of 1 mg/mL, just before intratracheal instillation. Supernatants for each NP were obtained by hard sedimentation (24,000 rpm, 15 h), with successful removal of 95–98% of NPs in solution; additional NPs characteristics, including particle characterization by TEM and zeta-potential at different pH can be found in [37,52,53], and a full discussion of the characterization methods and comparative results in [54].

### 4.2. Animals

Female, 6–10 weeks old BALB/c mice were obtained from Charles River (Sulzfeld/Germany), housed under specific pathogen free conditions in individually ventilated cages (VentiRack; Biozone, Margate/UK) and fed by standard diet and water ad libitum. The *in vivo* study was conducted under federal guidelines for the use and cares of laboratory animals and was approved by the Government of the District of Upper Bavaria and the Animal Care and Use Committee of the Helmholtz Center Munich. Nlrp3^−/−^ Mice [29] were housed at the Center for Preclinical Research, Klinikum rechts der Isar, Technical University of Munich. 

### 4.3. Cell Preparation and In Vitro Inflammasome Assays

Bone marrow-derived dendritic cells (BMDC) from WT and NLRP3^−/−^ mice were differentiated from tibial and femoral bone marrow aspirates, as previously described [29,55,56]. Cells were differentiated for seven days with growth factors recombinant mouse M-CSF (rmM-CSF) and recombinant mouse GM-CSF (rmGM-CSF) (Immunotools), and were then seeded in 96-well plates at a density of 10^6^ cells/mL. Cells were primed with 20 ng/mL *E. coli* K12 ultra-pure LPS (Invivogen) for 3 h and treated with inflammasome activators (300 µg/mL of MSU or alum) or silica NPs (0–1000 µg/mL) for 0.5–6 h. All stimulations were performed in triplets and cytokine production in cell-free supernatants was measured by ELISA.

For analysis of supernatants by Immunoblot, triplet samples were pooled and analyzed using standard techniques. For cytokine detection in cellular extracts, supernatants were removed and the cells were washed with PBS. Sodium Dodecyl Sulphate (SDS)-containing sample buffer was added directly to the cells. 

### 4.4. Nanoparticles Instillation In Vivo

To evaluate the immunomodulatory effect of NPs, a protocol of mild allergic inflammation in the lung was used, as previously described [21,36]. Briefly, mice were sensitized by repetitive intraperitoneal injections of 1 μg ovalbumin (OVA) (grade VI; Sigma-Aldrich, St Louis, MO, USA) in PBS adsorbed to 2.5 mg aluminum hydroxide (alum) (Thermo Fisher Scientific, Waltham, MA, USA) on days 0, 7, 14, 28, and 42. Blood samples were taken before and after sensitization. OVA/alum-sensitized mice (S mice), compared to non-sensitized mice (NS mice), were characterized by high titers of OVA-specific immunoglobulin E (8.05 ± 1.64 vs. 0.1 ± 0.03 μg/mL). At day 52, mice were intratracheally instilled with 50 μg (1 mg/mL) of SiO_2_ NPs, or with their correspondent supernatant control (CTL). NP instillation was followed by OVA aerosol challenge for 20 min with 1% OVA in PBS that was delivered by a PARI BOY nebulizer (PARI GmbH, Starnberg, Germany). Five days after OVA challenge, lungs were snap frozen in liquid nitrogen and stored at −80 °C until use.

### 4.5. Real-Time Polymerase Chain Reaction

Total RNA was extracted from snap-frozen lung tissue, as previously described [48]. For polymerase chain reaction (PCR) arrays (*n* = 4/group of experiment) (SABiosciences, Hilden, Germany), 0.4 μg RNA was converted into complementary cDNA with the RT2 First Strand Kit (Qiagen, Hilden, Germany) and real-time PCR was performed with RT2 SYBR Green ROX qPCR Mastermix (Qiagen) and ViiA™ 7 thermocycler (Life Technologies, Ober-Olm, Germany). Relative expression levels were calculated using the 2^ΔΔ*CT*^ method [57], normalized to the arithmetic mean of glyceraldehyde-3-phosphate dehydrogenase (GAPDH) and beta-actin (ACTB). Data were considered significant with a *p*-value ≤ 0.05 with a confidence interval of 95%. The array results indicated that supernatants from the four NPs were consistent, and hence are presented here as an average.

### 4.6. Transmission Electron Microscopy

Lungs were fixed by intratracheal perfusion with 2.5% glutaraldehyde in 0.1 M cacodylate buffer (pH 7.4), as previously described [43]; the peripheral lung was minced and immersed in the same fixative for 4–7 days at 4 °C, post fixed in 1% osmiumtetroxide in cacodylate buffer for 1 h at 4 °C and embedded in EMbed 812 (Science Services, München, Germany). After tissue evaluation on semithin sections, silver coloured ultrathin sections were mounted on formvar-coated 75-mesh nickel grids, double-stained with uranyl acetate and lead citrate and observed in a transmission electron microscope (Zeiss EM 10 C/CR, Oberkochen, Germany) at 80 kV. 

### 4.7. Statistical Analysis

Differences amongs groups were calculated with a one-way analysis of variance with Bonferroni post hoc test (GraphPad Prism; GraphPad Software, Inc., La Jolla, CA, USA). Statistically significant values were categorized as follows: *p* < 0.05, *p* ≤ 0.01, and *p* ≤ 0.001.

## 5. Conclusions

This study propose two surface modifications of silica NPs, amino, and phophonate, as successuful strategy to reduce the inflammasome activation and the pulmonary inflammation induced by uncoated SiO_2_ NPs, both *in vitro* and *in vivo*. It also shows that PEGylation, although it reduced inflammasome activation *in vitro*, was not a successful coating strategy to reduce the pulmonary inflammation milieu *in vivo*. This highlights the requirement of multiple assays to investigate the effect of surface modifications in limiting NPs inflammatory potential. 

When considering the stronger effect observed by plain/PEG NPs in sensitized mice, we might consider special protection for asthmatics that are exposed to NPs at the work place.

## Supplementary Materials

The following are available online at www.mdpi.com/2079-4991/7/11/355, Figure S1: Localization of SiO_2_ NPs in the lungs by transmission electron microscopy. Figure S2: Effect of sensitization on inflammasome activation and expression of inflammatory genes *in vivo*.
