# Supplementary material for: Specific Surface Modifications of Silica Nanoparticles Diminish Inflammasome Activation and In Vivo Expression of Selected Inflammatory Genes
Source: Nanomaterials (Basel). 2017 Oct 30;7(11):355. doi: 10.3390/nano7110355 (PMC5707572; doi:10.3390/nano7110355)
Supplement: Supplementary file 1 [file nanomaterials-07-00355-s001.pdf]

Supplementary Materials

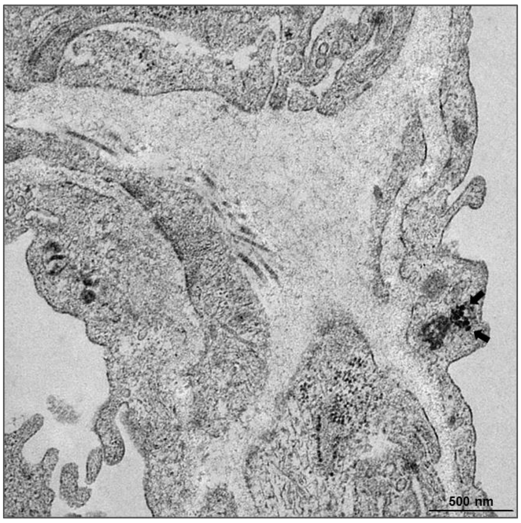

Figure S1. Localization of SiO_2 NPs in the lungs by transmission electron microscopy. Same epithelial type I cell from NS lungs exposed to SiO_2 plain as represented in Figure 7b, at higher magnification. Ultrastructural localization of SiO_2 NPs surrounded by a single membrane (arrows) is evident within the cell cytoplasm.

a **b**

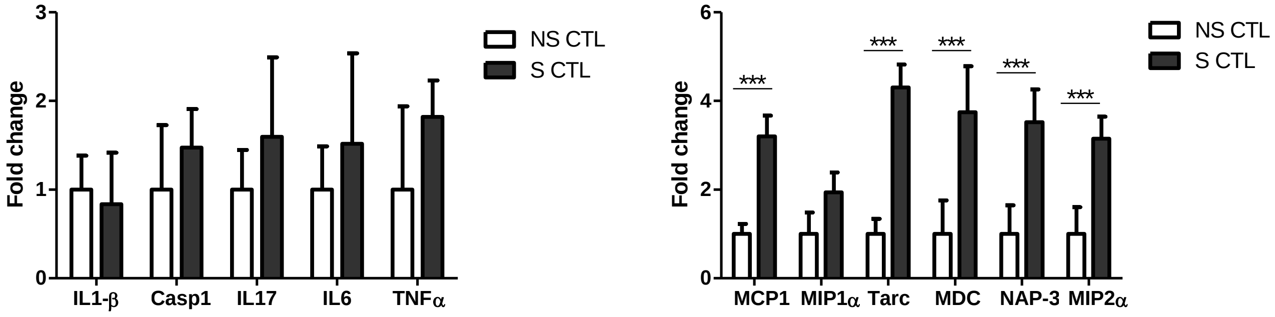

Figure S2. Effect of sensitization on inflammasome activation and expression of inflammatory genes *in vivo*. Gene expression was evaluated in NS and S mice 5 days after intratracheal instillation of 50 μg of NPs supernatant control (CTL) and ovalbumin challenge. Mean ± standard deviation were evaluated in the lung by real-time PCR (n=4/group). ***$p \leq 0.001$ vs NS, evaluated by two-way ANOVA.
